# Pristimerin induces apoptosis and inhibits proliferation, migration in H1299 Lung Cancer Cells

**DOI:** 10.7150/jca.44431

**Published:** 2020-09-02

**Authors:** Jiajun Li, Qiaoru Guo, Xueping Lei, Lingling Zhang, Chaoyue Su, Yun Liu, Wenmin Zhou, Hubiao Chen, Hui Wang, Fenghua Wang, Yanyan Yan, Jianye Zhang

**Affiliations:** 1 Guangdong Provincial Key Laboratory of Molecular Target & Clinical Pharmacology, School of Pharmaceutical Sciences and the Fifth Affiliated Hospital, Guangzhou Medical University, Guangzhou 511436, P. R. China.; 2 School of Chinese Medicine, Hong Kong Baptist University, Hong Kong, P. R. China.; 3 Guangzhou Institute of Pediatrics/Guangzhou Women and Children's Medical Center, Guangzhou Medical University, Guangzhou 510623, P. R. China.; 4 Institute of Immunology and School of Medicine, Shanxi Datong University, Datong 037009, P. R. China.

**Keywords:** Anti-lung cancer, NSCLC, pristimerin, anti-apoptosis, migration, invasion

## Abstract

**Background:** The natural occurring pristimerin, a quinonemethide triterpenoid, is extracted from a variety of species of the *Celastraceae* and *Hippocrateaceae* family. This research investigated the *in vitro* anti-cancer potential of pristimerin on NSCLC cells NCI-H1299 and elucidated the molecular mechanism.

**Methods:** Cell growth inhibition by pristimerin was assessed using the MTT assay. Apoptosis was detected using the Annexin V/propidium iodide (PI) test. The colony forming assay was used to investigate the anti-proliferative effects of pristimerin. Wound healing assay and the transwell cell migration assay were utilized to determine the inhibitory effects of migration and invasion, respectively. Western blot was used to detect the protein expression, and real-time-quantitative (RT-q) PCR was used to analyze the mRNA expression.

**Results:** The results showed that pristimerin inhibited the proliferation of H1299 cells with an IC_50_ value of 2.2 ± 0.34 µM and induced apoptosis in a dose-dependent manner. The colony formation ability was reduced in a dose-dependent manner. A marked inhibition of migration and invasion against H1299 cells was observed in a dose- or time-dependent manner. Moreover, the decreased protein levels of vimentin, F-actin, integrin β1, matrix metalloproteinase (MMP2) and Snail revealed the potential inhibition of epithelial-to-mesenchymal transition (EMT). The regulated mRNA levels of integrin β1, MMP2 and Snail indicated the great potential in the treatment of NSCLC.

**Conclusion:** In conclusion, our study demonstrated that pristimerin suppressed NSCLC cells NCI-H1299 *in vitro*, exhibited potent activities of proliferation inhibition and apoptosis induction. Furthermore, the treatment of pristimerin decreased migration and invasion of H1299, which was correlated with EMT-related proteins and mRNA.

## Introduction

Lung cancer is one of the most common malignant tumor throughout the world, accounting for the highest diagnostic rate (11.6%) and cancer death rate (18.4%), approximately 80% of which belongs to non-small cell lung cancer (NSCLC) 1, 2. Although the targeted molecular therapy and immunotherapy has made great progresses 3, until recently, for early-stage lung cancer patients, chemotherapy remained the non-operative first-line approach (other than radiofrequency ablation and standard radiotherapy) 4. Traditional chemotherapeutic drugs are still faced with drug-resistance and related-toxicities and active development of new anti-cancer agents is warranted and promising.

Natural product contains abundant resources of bioactive agents, the anti-cancer activities of natural product which attracts increasing attention 5-8. The natural occurring pristimerin, a quinonemethide triterpenoid, is isolated from varied *Celastraceae* and *Hippocrateaceae* family 9-11. Recently, pristimerin has been reported to widely induce apoptosis and autophagy, inhibit angiogensis and metastasis against cancer 12. However, the potential efficacy of pristimerin against lung cancer is relatively deficient. In this article, we investigated the *in vitro* anti-cancer potential of pristimerin on NSCLC cells NCI-H1299 and elucidated the molecular mechanism.

## Materials and Methods

### Cell culture and reagents

**Figure 1:**
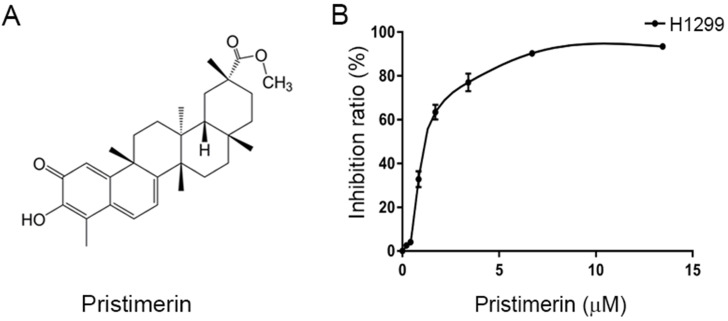
Chemical structure of pristimerin and its ability to inhibit NCI-H1299 cells proliferation. **A.** Chemical structures of pristimerin. **B.** Pristimerin shows potent cytotoxicity to H1299 cells measured by MTT assay. The cells having grown for 24 h were exposed to a full range of concentrations of pristimerin for 72 h.

The human lung adenocarinoma epithelial cell line NCI-H1299 (lack expression of p53 protein) was obtained from iCell Bioscience Inc., Shanghai, China, and authenticated by short-tandem repeat (STR) profiling. The cells were maintained in RPMI-1640 medium supplemented with 100 μM streptomycin, 100 U/mL penicillin and 10% fetal bovine serum (FBS) (TIANHANG Biotech Co., Ltd., Zhejiang, China). Cells were cultured in a humidified atmosphere containing 5% CO_2_ at 37°C. Pristimerin (Fig. 1A) with a purity of >98% was purchased from the Spring & Autumn Biological Engineering Co., Ltd. (Nanjing, China).

### Cell viability assay

The MTT assay was utilized to evaluate the cytotoxic activity of pristimerin. 4 × 10^3^ cells were transferred into each well of 96-well plates in a final volume of 190 μL. After cells attached for 24 h, 10 μL pristimerin were added to the medium at a series of different concentrations for 72 h incubation. 20 μL MTT (5 mg/mL) was added to each well for 4 h reaction. Next, the supernatant was discarded, and the formed formazan product was dissolved in 100 μL DMSO/well. The optical density (OD) was detected at an absorbance wavelength of 540 nm using a Model 550 Microplate reader (Bio-Rad Laboratories, Berkeley, CA, USA), with reference filter of 655 nm. The IC_50_ value was used to express the inhibitory action of pristimerin, which was obtained according to the concentration-response curve using Bliss software. Percent survival rate was obtained using the formula as described previously 5.

### Annexin V/ propidium iodide (PI) test

Annexin V-FITC/PI double staining assay was applied to determine apoptosis, using an Annexin V-FITC/PI apoptosis detection kit (KeyGen Biotech Co., Ltd., Nanjing, China). H1299 cells were cultured in the presence of 0.9, 1.8 and 3.6 μM pristimerin for 48 h, then collected and resuspended in 0.5 mL binding buffer/sample for 5 min. Subsequently, 5 μL FITC-labeled Annexin-V and 5 μL PI were added for 15 min staining at 37°C in dark. The stained samples were measured using a flow cytometer (Beckman Coulter, Brea, CA, USA) and analyzed by CytExpert software version 2.2 (Beckman Coulter, Brea, CA, USA). The rate of apoptosis (%) was determined by dividing the number of apoptotic cells by the number of total cells observed, and multiplying with 100 7. All experiments were repeated three times.

### Colony forming assay

Cells were split and transferred into 12-well plates with 400 cells/well. Until the colonies of 30-40 cells formed, cells were incubated with pristimerin of 1.8 μM for 24, 48 and 72 h, respectively. The wells were washed three times with PBS, then fixed with methanol and stained with 0.5% (w/v) crystal violet. Images were captured using ChemiDoc™ XRS+ (Bio-Rad Laboratories, Berkeley, CA, USA). The colony forming assay was repeated three times in duplicate.

### Wound healing assay

Cells were digested using trypsin and resuspended in serum-free medium. The cells were transferred into 6-well plates and grown to reach confluence overnight. Subsequently, linear wounds were scraped with pipette tips in the cell monolayer. Next, the debris was removed and the edge of the wound was smoothed by washing the cells once. The cells were incubated with pristimerin (0.9, 1.8 and 3.6 μM), respectively. After 0, 24 and 48 h of incubation, wounds were observed and photographed using a light microscope (Olympus Life Science, Tokyo, Japan) at ×40 magnification. Cell motility was quantified by the percentage of the repaired area. The percent wound closure (%) was obtained through the following formula: percent mobility rate = migrated cell surface area/total surface area × 100 13.

### Invasion assay

The bottom of transwell apparatus (Corning Costar, Corning, NY, USA) was pre-coated with matrigel (Corning Costar, Corning, NY, USA), which was diluted by 5 times with basal medium. Cells were transferred into the upper chamber of the transwell apparatus at a density of 8 × 10^3^ cells/well in 200 μL of serum-free RPMI-1640 medium, which contained the pristimerin (0.9, 1.8 and 3.6 μM). The lower chamber contained 600 μL RPMI-1640 medium adding 10% FBS, which was a chemoattractant. After incubation of 48 h, cells that migrated into the lower chamber were fixed with methanol and stained with 0.5% (w/v) crystal violet 14. Cells on the top layer were removed using a cotton swab, and the migrated cells were photographed using an inverted microscope (Leica Microsystems, Wetzlar, Germany) at ×200 magnification. Nine random fields were acquired to quantify attached and migrated cells. The cell number was quantified by IMAGE-J software (version 1.51j8, Bethesda, MD, USA) (http://imagej.nih.gov/ij/). Cell counts were expressed as the mean number of cells per field of view.

### Western blot

Cultured cells from different samples were collected and washed twice with ice-cold PBS. The pellet was vortexed and lysed in lysis buffer (Cell Signaling Technology, Danvers, MA, USA), supplementary with Proteinase Inhibitor Cocktail (Cell Signaling Technology, Danvers, MA, USA) and phenylmethylsulfonyl fluoride (PMSF) (Cell Signaling Technology, Danvers, MA, USA). 25 μg/lane proteins were separated by sodium dodecyl sulfate polyacrylamide gel electrophoresis (SDS-PAGE) and subsequently transferred onto PVDF membranes (Millipore, Boston, MA, USA). The membranes were then blocked with TBST buffer containing 5% dried non-fat milk, subsequently incubated with primary antibodies overnight at 4°C. After 3 times washed with TBST buffer, the membranes were incubated to bind horseradish peroxidase (HRP) conjugated secondary antibodies for 2 h at room temperature. Blots were visualized by Western Lightning^®^ Plus-ELC kit (PerkinElmer, Waltham, Ma, USA) and exposed by ChemiDoc™ XRS+ (BIO-RAD, Berkeley, CA, USA). Antibodies used in this study included: rabbit anit-Integrin beta 1 (1:1000 dilution) (#9699, Cell Signaling Technology), rabbit anit-MMP2 (1:1000 dilution) (ab92536, Abcam), rabbit anit-vimentin (1:1000 dilution) (#5741, Cell Signaling Technology), mouse anti-F-actin (0.2 µg/mL) (ab4792, Abcam), rabbit anti-Snail+Slug (1:1000 dilution) (ab180714, Abcam), mouse anti-GAPDH (1:10000 dilution) (MB001, Bioworld Technology), GAPDH (1A6) monoclonal antibody (1:10000 dilution) (MB001, Bioworld Technology), Anti-rabbit IgG, HRP-linked Antibody (1:2000 dilution) (#7074, Cell Signaling Technology) and HRP-conjugated goat anti-mouse (1:5000 dilution) (BS12478, Bioworld Technology).

### mRNA extraction, reverse transcription and real-time-quantitative (RT-q) PCR

Total RNA of H1299 cells stimulated with pristimerin (0.9, 1.8, 2.7 and 3.6 μM) after 24 h was extracted using TRIzol (Thermo, Waltham, MA, USA). The PrimeScript™ RT Master Mix (Takara Biomedical Technology, Kusatsu, Japan) was used to transcribe mRNAs into cDNA. The forward and reverse primers (Takara Biomedical Technology, Kusatsu, Japan) were designed to detect the expression of integrin β1, matrix metallopeptidase 2 (MMP2) and Snail. SYBR^®^ Premix Ex Taq™ (TliRNaseH Plus) (Takara Biomedical Technology, Kusatsu, Japan) was utilized for RT-qPCR and GAPDH was used to normalize mRNA expression. RT-qPCR was carried out using a QuantStudio 5 (Thermo Fisher scientific, Waltham, MA, USA). The program setting for qPCR is as below: firstly, initial denaturation at 95°C for 30 sec; then 40 reaction cycles were followed by denaturation at 95°C for 5 sec, annealing at 60°C for 34 sec, and then elongation at 72°C for 30 sec 15. The data were calculated using the 2^‑ΔΔCq^ method and GAPDH was used as control gene for normalization 16. The primer sequences designed are listed in Table 1.

### Statistical analysis

All experiments were performed in triplicate unless otherwise stated. The differences between groups were analyzed using ANOVA followed by Tukey's post-hoc test. Statistical differences in each assay were analyzed by SPSS software version 16.0 (SPSS, Inc., Chicago, IL, USA). Differences between groups were considered statistically significant if *P* < 0.05.

## Results

### Pristimerin exhibits a notable cytotoxicity against H1299 cells

Cell growth inhibition by pristimerin was assessed using MTT assay. Pristimerin (Fig 1A) inhibited the proliferation of H1299 cells with an IC_50_ value of 2.2 ± 0.34 μM. The growth inhibition occurred in a dose-dependent manner (Fig. 1B). The results indicated the cytotoxicity of pristimerin against H1299 cells.

### Pristimerin shows a potent apoptotic-inducing effect against H1299 cells

**Figure 2:**
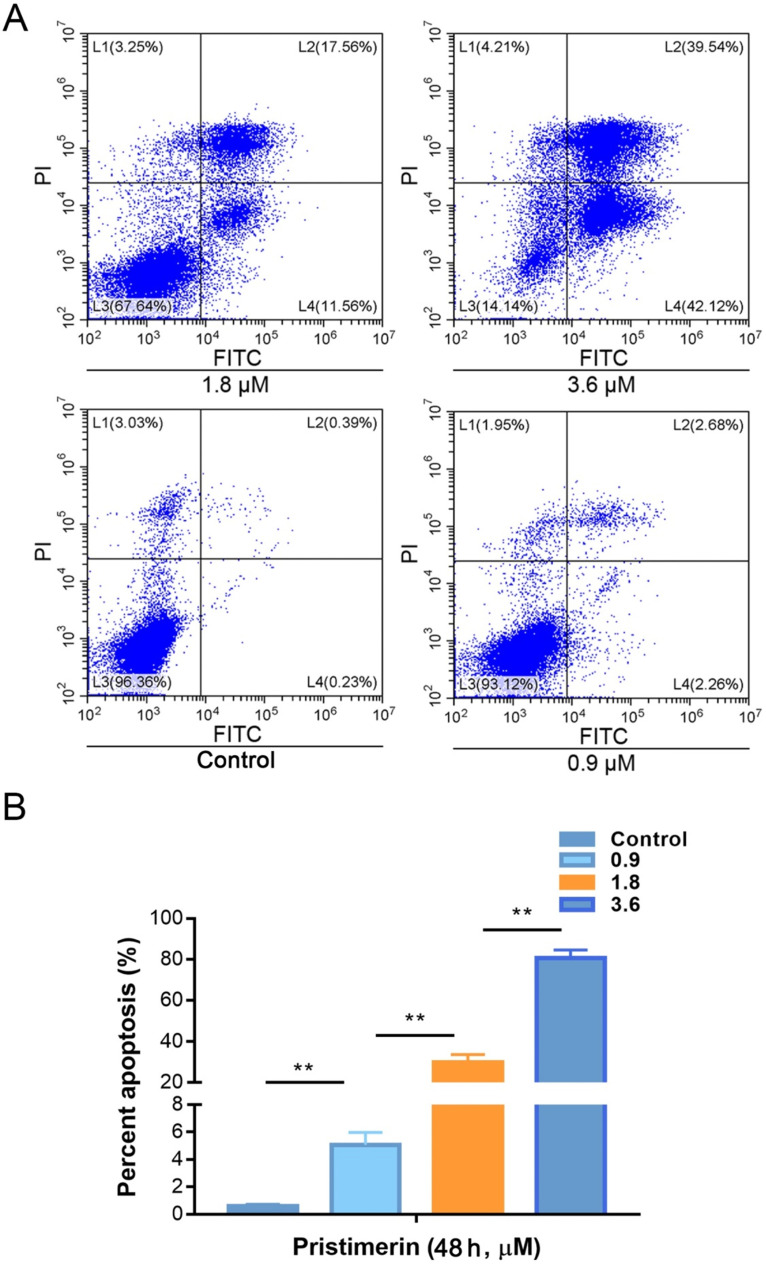
Pristimerin induces lung cancer H1299 cells apoptosis *in vitro*. **A.** The lung cancer H1299 cells were treated with pristimerin for 48 h and apoptosis were detected by FACS. **B.** Quantitative results of (A). Data were quantified as mean ± standard deviation. **P < 0.01, n ≥ 3.

To investigate whether pristimerin is able to induce apoptosis of H1299 cells. The cells were incubated with 0.9, 1.8 and 3.6 μM of pristimerin for 48 h, then apoptosis was detected by flow cytometry based on annexin V and PI staining to discriminate healthy cells from early/late apoptotic cells and necrotic cells. The results showed that the rates of apoptosis were 0.61 ± 0.11, 5.08 ± 0.90, 29.79 ± 0.88 and 80.64 ± 3.92% for control, 0.9, 1.8 and 3.6 μM of pristimerin, respectively (Fig. 2).

### Pristimerin decreases H1299 cells colony formation

**Figure 3:**
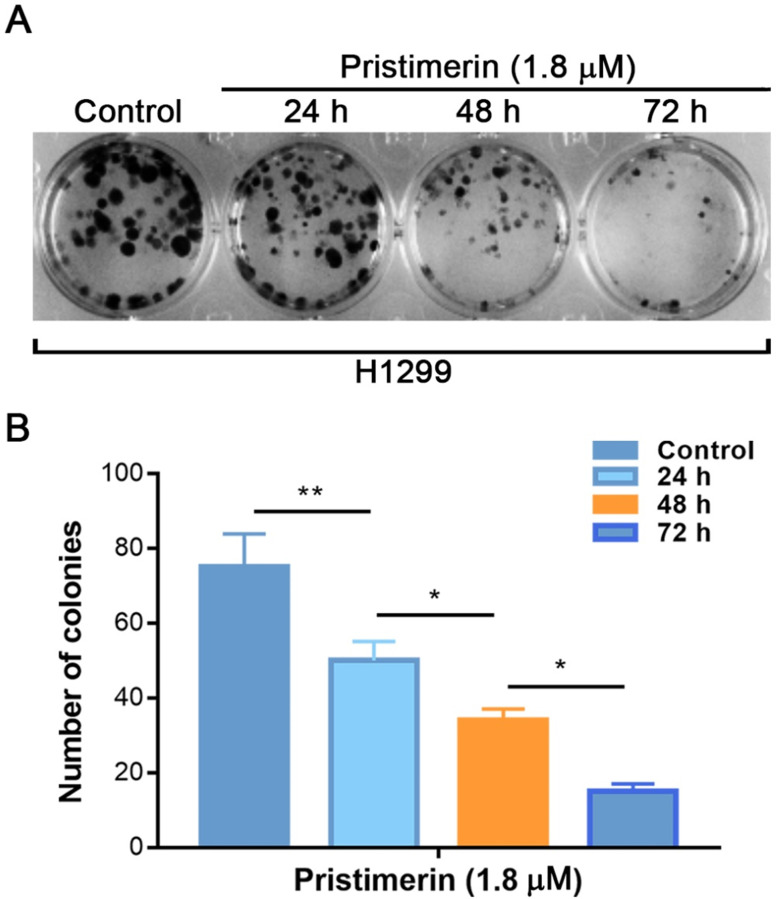
Pristimerin of 1.8 µM leads to a significant inhibition of colony formation of H1299 cells in a time-dependent manner. **A.** H1299 cells were treated with different concentrations of pristimerin and colony efficiency was observed *via* a colony forming cell assay. **B.** Quantitative results of (A). Data were quantified as mean ± standard deviation. *P < 0.05, **P < 0.01, n ≥ 3.

The colony forming assay was performed to further investigate the anti-proliferative effects of pristimerin. The results showed that incubation of H1299 cells with pristimerin for 24, 48 and 72 h reduced the colony formation in a time-dependent manner which was characterized by specific colony numbers and sizes of H1299 cells (Fig. 3).

### Pristimerin reduces H1299 cells migration and invasion

**Figure 4:**
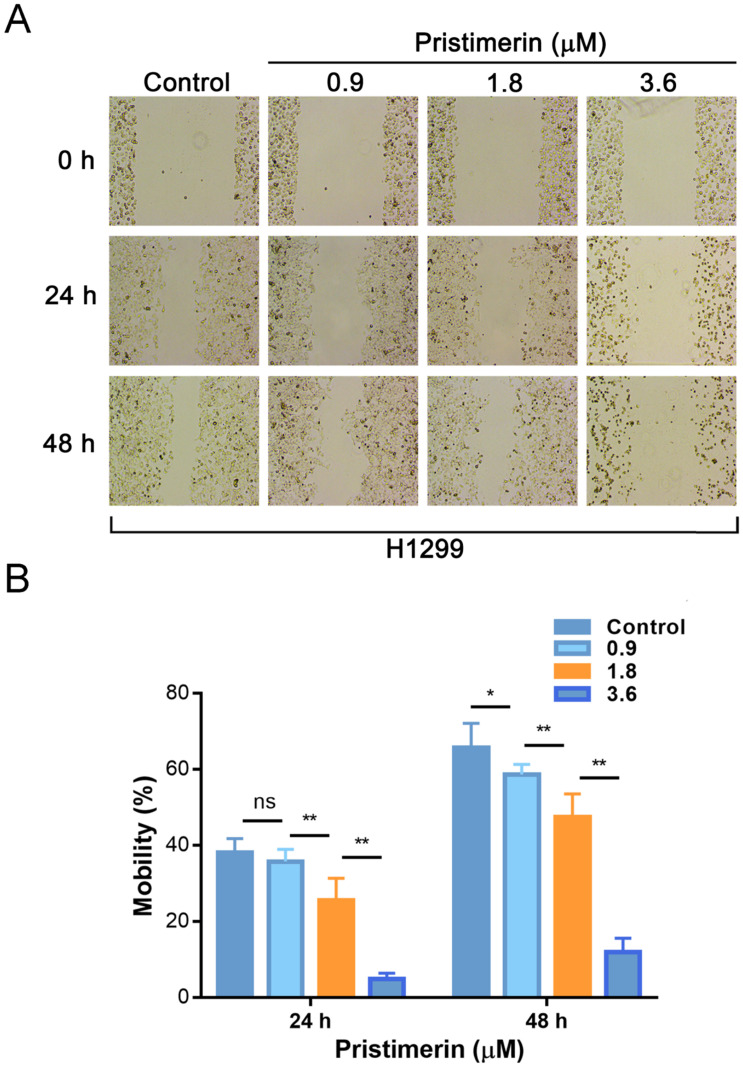
Treatment of pristimerin decreases the migrative ability of H1299 cells *in vitro* in a dose-dependent manner. **A.** H1299 cells were treated with different concentrations of pristimerin for 24 h and 48 h, the mobility ratio was observed *via* wound healing assays. **B.** Quantitative results of (A). Data are presented as the mean ± standard deviation. *P < 0.05, **P < 0.01, n ≥ 3.

Wound healing assay was applied to evaluate the inhibitory effect of pristimerin on NSCLC cells migration. After H1299 cells were exposed to pristimerin for 24 h at a concentration of 0.9, 1.8 and 3.6 μM, the mobility ratios with respect to control were 38.03 ± 3.71, 35.67 ± 3.23, 25.54 ± 5.76 and 4.91 ± 1.47%, respectively. Moreover, after exposure to pristimerin for 48 h at the same series of concentration, the mobility ratios were 65.66 ± 6.42, 58.61 ± 3.72, 47.36 ± 6.18, 11.92 ± 3.63%, respectively (Fig. 4). The result suggested that pristimerin markedly inhibited H1299 cells migration in a dose-dependent manner.

**Figure 5:**
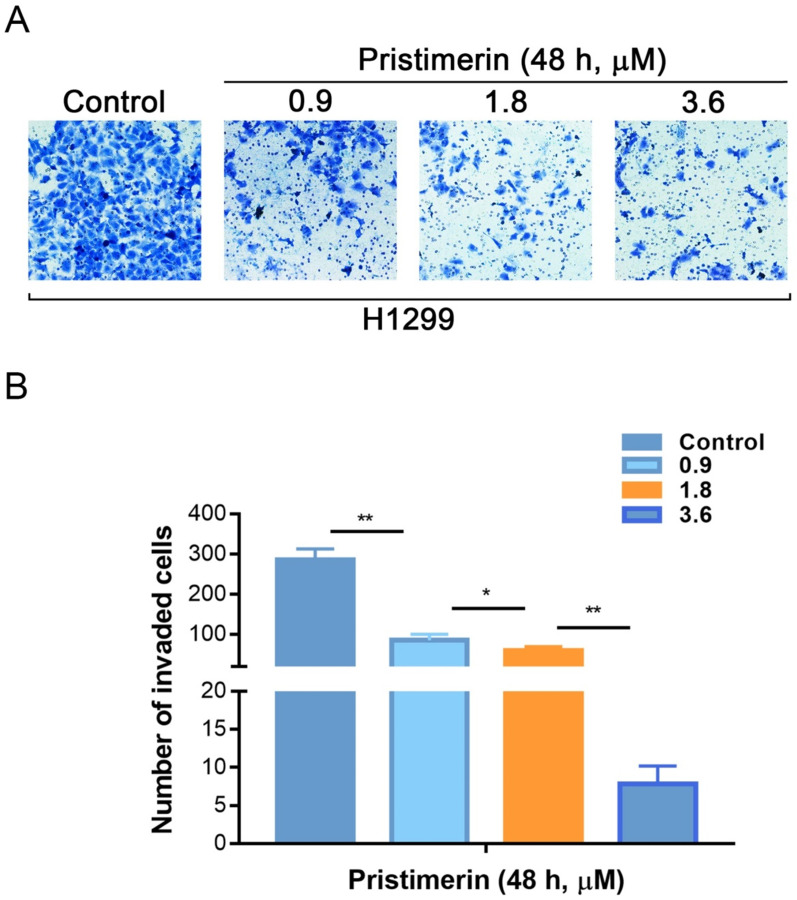
H1299 cells invasion is inhibited *in vitro* by pristimerin in a dose-dependent manner. **A.** The invasive ability of H1299 cells was detected *via* transwell assay. Following treatment of H1299 cells with different concentrations of pristimerin for 48 h, the number of invaded cells decreased in a dose-dependent manner. **B.** Quantitative results of (A). Data are presented as the mean ± standard deviation. *P < 0.05, **P < 0.01, n ≥ 3.

Invasion is another process of cancer proliferation and metastasis. Transwell assay was utilized to analyze the role of pristimerin in regulating the invasion of NSCLC cells. Consistent with the results of migration assay, pristimerin markedly inhibited NSCLC cells invasion. After treatment of H1299 cells with 0.9, 1.8 and 3.6 μM pristimerin for 48 h, the number of invaded cells decreased (Fig. 5). The above results indicated that pristimerin reduced cell invasion in a dose-dependent manner.

### Pristimerin regulates protein expression

**Figure 6:**
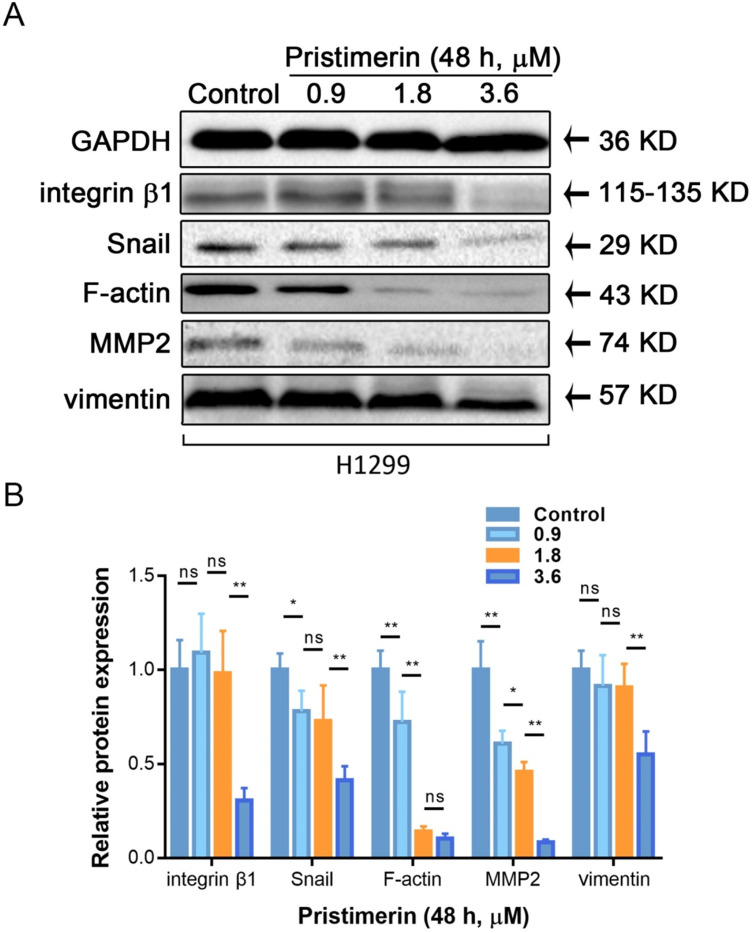
Pristimerin regulates migration and invasion-related proteins expression in H1299 cells. After H1299 cells were treated with pristimerin of 0.9, 1.8 and 3.6 μM for 48 h respectively, the whole-cell lysates were assayed by Western blot and corresponding antibodies. **A.** Down-regulations of vimentin, F-actin, integrin β1, MMP2 and Snail expression by pristimerin in a dose-dependent manner were observed. **B.** Data analysis of (A). Data are presented as the mean ± standard deviation. *P < 0.05, **P < 0.01, n ≥ 3.

Cancer metastasis is a complicated process, which involved motility, adhesion and disruption of polarity of cell. To investigate whether pristimerin inhibited EMT of H1299 cells, western blot was used to detect the protein express of vimentin, F-actin integrin β1, MMP2 and Snail. After H1299 cells were incubated with 0.9, 1.8 and 3.6 μM pristimerin for 48 h, the protein expression of vimentin, F-actin, integrin β1, MMP2 and Snail were decreased in a dose-dependent manner (Fig. 6).

### Pristimerin affects mRNA expression

**Figure 7:**
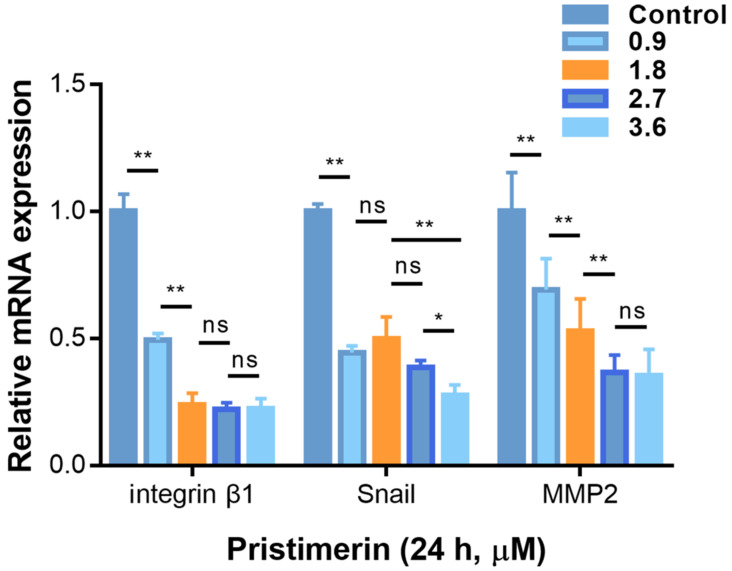
Pristimerin regulates migration and invasion-related mRNA in H1299 cells. **A.** After H1299 cells were treated with pristimerin of 0.9, 1.8, 2.7 and 3.6 µM for 24 h respectively, the mRNA expression was detected by RT-qPCR. Down-regulation of Snail, and integrin β1and MMP2 mRNA expression were observed. **B.** Data analysis of (A). Data are presented as the mean ± standard deviation. *P < 0.05, **P < 0.01, n ≥ 3.

To determine whether the anti-proliferative, anti-migration and anti-invasive activities of pristimerin were related to the specific mRNA levels, RT-qPCR was used to determine the mRNA expression. After H1299 cells were incubated with pristimerin (0.9, 1.8, 2.7 and 3.6 μM) for 24 h, the mRNA levels of integrin β1, MMP2 and Snail were down-regulated in a dose-dependent manner (Fig. 7).

## Discussion

Pristimerin (Fig. 1A), a quinonemethide triterpenoid, kills cancer cell lines in a wide spectrum 12, as well as potent *in vivo* anti-cancer activities 17, 18. However, its activities against lung cancer are insufficience of evidence to support. It was reported that pristimerin has shown activities of proliferation-inhibiting and apoptosis-inducing in NSCLC cells 11, 19. At the present research, pristimerin exhibited a powerful cytotoxicity to H1299 cells, with IC_50_ value of 2.2 ± 0.34 μM (Fig. 1B). Apoptosis and autophagy induced by pristimerin were widely revealed 20-22. In our study, the annexin V-FITC/PI test was used to detect apoptosis. The results showed that apoptosis was strongly induced in a dose-dependent manner in H1299 cells (Fig. 2). Subsequently, colony forming cell assay was utilized to detect the anti-proliferative ability of pristimerin. The decreased cell numbers and sizes of H1299 cells showed that pristimerin time-dependently inhibited cell proliferation (Fig. 3).

Cancer metastasis is the most common reason that caused human cancer deaths, while only a minority of lung cancers are localised when diagnosis 23-26. In order to investigate whether pristimerin inhibited motility of NSCLC cells, wound healing assay and transwell assay were applied. The experimental data indicated that pristimerin significantly decreased NSCLC cells migration in a dose-dependent manner (Fig. 4), as well as mobility (Fig. 5).

Western blot assay was subsequently utilized to find out the regulated protein targets related to this process. Vimentin controls the alignment of the cell traction forces to direct single-cell mesenchymal migration, and marks epithelial-mesenchymal transition (EMT) 27. As the data showed, vimentin was dose-dependently down-regulated. Additionally, F-actin, another cytoskeleton polymers needed for migration 28, was also decreased by pristimerin. To complete cell adhesion, cellular cytoskeleton needs the assistant of integrin family to connect extracellular matrix (ECM) network 29, the decreased integrin β1 in our results indicated the inhibition of adhesive process 30. Besides the cancer cell-cell adhension, it also needs the ability to destroy the histological barrier of cancer cell invasion. Matrix metallopeptidase (MMP) is considered as an important member of the invasive process, which cancer cell secreted to dissolve the components of ECM or basement membrane 31. It was previously reported that pristimerin down-regulated MMP2 and matrix metallopeptidase 9 (MMP9) to inhibit migration and invasion of esophageal squamous cell carcinoma (ESCC) 32. Similarly, we found an obviously decreased expression of MMP2 in our study. Zuo *et al.* demonstrated the inhibitory action on EMT activation in prostate cancer (PCa) PC-3 cells 33, which was confirmed by EMT markers including N-cadherin, fibronectin, vimentin and ZEB1. While in our study, the activities of pristimerin to reduce the cellular cytoskeleton of vimentin and F-actin, cellular adhesion of integrin β1, as well as cellular invasion of MMP2, exerted the multiple ways of anti-cancer potential. Furthermore, the key EMT upstream protein Snail was inhibited revealed the relationship of this process (Fig. 6) 34.

**Figure 8:**
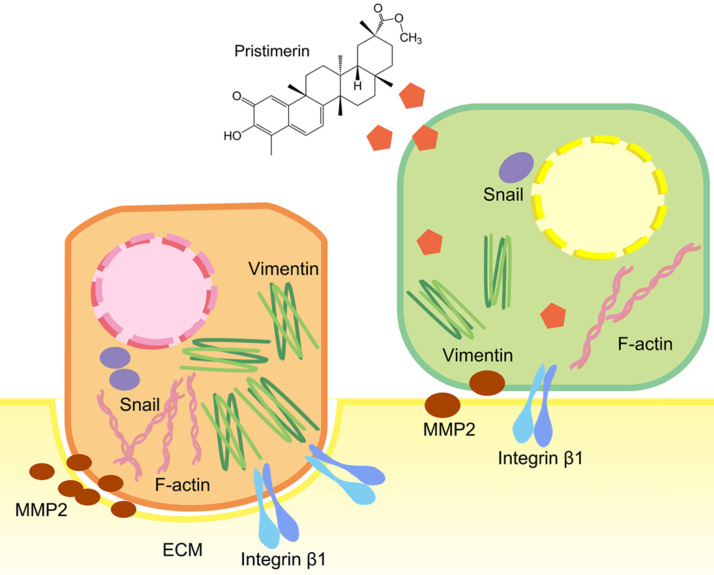
Brief summary of molecular mechanisms involved in pristimeirn-induced anti-proliferative, anti-migration and anti-invasive effects against human NSCLC cell line NCI-H1299.

To further explore whether the inhibition of these proteins was corelated with mRNA levels, RT-qPCR was applied. Consistent with the protein levels, integrin β1, MMP2 and Snail were down-regulated in a dose-dependent manner after 24 h treatment. This indicated pristimerin may regulate integrin β1, MMP2 and Snail proteins *via* suppressing their mRNA translation (Fig. 7). The above molecular mechanisms of pristimerin are summarized in Fig. 8.

## Conclusion

In conclusion, our study demonstrated that pristimerin suppressed NSCLC cells NCI-H1299 *in vitro*, exhibited potent activities of proliferation inhibition and apoptosis induction. Furthermore, the treatment of pristimerin decreased migration and invasion of H1299 cells, which was correlated with EMT-related proteins and mRNA.

## Figures and Tables

**Table 1:** The primer sequences employed in RT-qPCR

Primer name	Primer sequence (5'--3')	Company
GAPDH Forward	GGAAGGTGAAGGTCGGAGTCA	Invitrogen, Inc.
GAPDH Reverse	GTCATTGATGGCAACAATATCCACT	Invitrogen, Inc.
Integrin β1 Forward	GACGCCGCGCGGAAAAGATG	TaKaRa, Inc.
Integrin β1 Reverse	GCACCACCCACAATTTGGCCC	TaKaRa, Inc.
Snail Forward	CCAGACCCACTCAGATGTCAAG	TaKaRa, Inc.
Snail Reverse	GGGCAGGTATGGAGAGGAAGA	TaKaRa, Inc.
MMP2 Forward	CTCATCGCAGATGCCTGGAA	TaKaRa, Inc.
MMP2 Reverse	TTCAGGTAATAGGCACCCTTGAAGA	TaKaRa, Inc.
